# Myc-associated zinc-finger protein promotes clear cell renal cell carcinoma progression through transcriptional activation of the MAP2K2-dependent ERK pathway

**DOI:** 10.1186/s12935-021-02020-9

**Published:** 2021-06-28

**Authors:** Li-Xin Ren, Jin-Chun Qi, An-Ning Zhao, Bei Shi, Hong Zhang, Dan-Dan Wang, Zhan Yang

**Affiliations:** Department of Urology, The Second Hospital of Hebei Medical University, 215 Heping West Road, Shijiazhuang, 050000 China

**Keywords:** MAZ, MAP2K2, ERK, Signaling pathway, Clear cell renal carcinoma

## Abstract

**Background:**

The dysfunction of myc-related zinc finger protein (MAZ) has been proven to contribute to tumorigenesis and development of multiple cancer types. However, the biological roles and clinical significance of MAZ in clear cell renal carcinoma (ccRCC) remain unclear.

**Methods:**

MAZ expression was examined in ccRCC and normal kidney tissue by quantitative real-time PCR and Western blot. Statistical analysis was used to evaluate the clinical correlation between MAZ expression and clinicopathological characteristics to determine the relationship between MAZ expression and the survival of ccRCC patients. The biological roles of MAZ in cells were investigated in vitro using MTT and colony assays. Luciferase reporter assays and chromatin immunoprecipitation (ChIP) were used to investigate the relationship between MAZ and its potential downstream signaling molecules.

**Results:**

MAZ expression is elevated in ccRCC tissues, and higher levels of MAZ were correlated with poor survival of patients with ccRCC. MAZ upregulation elevates the proliferation ability of ccRCC cells in vitro, whereas silencing MAZ represses this ability. Our results further reveal that MAZ promotes cell growth, which is dependent on ERK signaling. Importantly, we found that MAZ positively regulates MAP2K2 expression in ccRCC cells. Mechanistically, MAZ binds to the MAP2K2 promoter and increases MAP2K2 transcription. Furthermore, MAP2K2 levels were shown to be increased in ccRCC tissues and to be associated with a poor prognosis of ccRCC patients. MAP2K2 upregulation activates the ERK signaling pathway and promotes ccRCC progression.

**Conclusion:**

These results reveal that the MAZ/MAP2K2/ERK signaling axis plays a crucial role in promoting ccRCC progression, which suggests the potential therapeutic utility of MAZ in ccRCC.

**Supplementary Information:**

The online version contains supplementary material available at 10.1186/s12935-021-02020-9.

## Background

Renal cell carcinoma (RCC) accounts for approximately 4% of all newly diagnosed cancers, and ranks sixth among male cancers and ninth among female cancers. In 2021, a total of 76,080 new RCC cases and 13,780 new deaths were reported in the United States [1]. Clear cell renal cell carcinoma (ccRCC) is the most common histological subtype of RCC, accounting for approximately 80% of adult clinical cases [2, 3]. Early-stage or local ccRCC is often treated with partial or radical nephrectomy [4, 5]. However, some ccRCC patients show symptoms only when they have distant metastases, and the 5-year survival rate of these patients is often less than 20% [6]. It has been confirmed that many genes are involved in the progress of ccRCC [7, 8], but the underlying molecular mechanism is still unclear. Therefore, the identification of molecular mechanisms is needed to understand the development of ccRCC and identify therapeutic targets in ccRCC.

Myc-related zinc finger protein (MAZ) is encoded by a 2.7 kb gene on chromosome 16p11.2 and is a protein composed of 477 amino acids [9]. As a transcription factor, MAZ can be combined with c-MYC and GA box (GGGAGGG) to regulate the initiation and termination of transcription [9, 10]. Report shows that MAZ is commonly expressed in human tissues, and its expression level varies according to the type of tissue [11]. A large number of studies have found that MAZ plays a vital role in the progression of a variety of cancers including breast, prostate and pancreatic cancer [11–13]. In prostate cancer, upregulated-MAZ promotes bone metastasis through the transcriptional activation of the KRas-dependent RalGEFs pathway [11]. By targeting MAZ, the circ-CUX1/EWSR1 regulation axis can inhibit glycolysis and neuroblastoma progression [14]. The MAZ, which is a downstream gene of the oncoprotein Cyr61/CCN1, promotes pancreatic cancer cell migration and invasion via CRAF-ERK signaling [12]. MAZ also regulates the pro-inflammatory response of colitis and colon cancer through STAT3 signaling [15]. However, the clinical significance and biological function of MAZ in ccRCC remain unclear.

MAP2K2, also known as MEK2 and MAPKK2, is a dual-specificity protein kinase that belongs to the MAP kinase kinase family, which also includes Map2k1 (MEK1). MEK1 and MEK2 share 79% amino acid identity and can also phosphorylate ERK substrates [16]. One study revealed that the MEK/ERK (MAPK) signaling cascade plays an essential role in mediating intercellular and intracellular communication, which regulates fundamental cell functions, such as growth, survival, and differentiation [17]. MAP2K2 controls activation of the MKK3/MKK6-p38 axis, which is involved in breast cancer cell survival by regulating cyclin D1 expression [18]. A recent study reported on the significance of the RAS/RAF/MEK/ERK signaling cascade, particularly with respect to its impact on clinical cancer therapy [17]. In ccRCC, circular RNA DHX33 promotes malignant behavior by targeting the miR-489-3p/MEK1 axis [19]. RKTG inhibits angiogenesis by suppressing MAPK-mediated autocrine VEGF signaling and is downregulated in ccRCC [20]. However, MAP2K2 expression in ccRCC and its underlying molecular mechanism with regard to cell proliferation remain unclear.

In the present study, we observed increased levels of MAZ in ccRCC tissue, and higher levels of MAZ or MAP2K2 were correlated with poor patient survival. Moreover, MAZ overexpression induced ccRCC cell proliferation in vitro. Importantly, we demonstrated that the MAP2K2/ERK pathway was involved in MAZ-promoted ccRCC cell growth. In addition, MAZ was shown to bind directly to the MAP2K2 promoter and to positively regulate MAP2K2 expression by transcriptional activation. Taken together, our results indicate that the MAZ/MAP2K2/ERK pathway plays a key role in the proliferation mechanism of ccRCC and provides a potential therapeutic target for ccRCC treatment.

## Methods

### Clinical samples

Human primary clear cell renal cell carcinoma and corresponding normal kidney tissue were collected from the Department of Urology, the Second Hospital of Hebei Medical University. All patients were ccRCC patients from July 2015 to June 2020. All patients underwent radical nephrectomy for treatment. The research protocol has been approved by the Ethics Committee of the Second Hospital of Hebei Medical University, and each patient’s written consent has been obtained.

### Cell lines and transfection

Human ccRCC cell lines (SW839, A498, Caki-1, 786-0) were purchased from ATCC (Rockville, Maryland, USA), and are preserved in our laboratory. The 293A cell line is deposited in our laboratory. The above-mentioned cells were cultured in low-sugar dulbecco’s modified eagle medium (DMEM) supplemented with 10% fetal bovine serum (Clark Bio, Claymont, DE, USA) and 1% penicillin/streptomycin (Solarbio, Beijing). The cells are cultured in a humidified condition of 95% air and 5% CO_2_. Cell transfection was performed using Lipofectamine 2000 (Invitrogen) according to the manufacturer’s operating manual.

### RNA isolation and qRT-PCR

Use RNAeasy Mini Elute Kit (QIAGEN) according to the manufacturer’s manual for total RNA isolation. The NanoDrop 2000 system detects RNA concentration and quality. The first strand of cDNA was synthesized using M-MLV First Strand Kit (Life Technologies) with random hexamer primers. Dilute the first strand of cDNA 5–10 times as needed, and use Platinum SYBR Green qPCR Super Mix UDG kit (Invitrogen) to perform real-time quantitative PCR (qRT-PCR) analysis on mRNA in ABI 7500 FAST System (Life Technologies). The relative transcriptional expression level of gene mRNA was standardized with GAPDH as an internal reference gene. The calculation was carried out using the 2^−ΔΔCt^ formula according to the previous description [21]. The primers used were as follows: MAZ-F: GAAGAACCATGCCTGCGAGATGTG; MAZ-R: GCTGCCTCACATTTCTCACATTTGAAG; MAP2K2-F: CGGTCACGGGATGGATAGC; MAP2K2-R: AGGGTTTTACACAACCAGCC; MAP2K1-F: CTTCGCAGAGCGGCTAGGAG; MAP2K1-R: TGGTCCCGTTAACTGCAGAG; MAPK1-F: TAAGGTGCCATGGAACAGGC; MAPK1-R: CCTCCAAACGGCTCAAAGGA; MAP3K10-F: GAGAACCACAACCTCGCAGA; MAP3K10-R: TCGATCTCACGGTAGGGGAC; MAP3K11-F: TCAACTGGGCTGTGCAGATT; MAP3K11-R: TGTCGTCACTCTCAATGGGC; MAP3K12-F: AGGGCCAGCTGTATGAGGTA; MAP3K12-R: AGGTAGTTCATGCCACCAGC; MAP7D1-F: ACCAAGAAGCAGGACAGCAA; MAP7D1-R: ATGGGCTCCTCTTTCTGCAC.

### Western blot analysis

Western blot of proteins in cells or tissues was performed as previously described [22]. The cultured cells and frozen tissue samples were used for protein extraction with RIPA lysis buffer. A modified Bradford method was used for protein quantitative analysis and the same amount of protein was loaded onto the gel. Then SDS-PAGE was performed to separate the proteins. Electrotransfer the protein from SDS-PAGE to a polyvinylidene fluoride (PVDF) membrane (Millipore). The membrane was sealed with 5% milk for 2 h. Finally, incubate the membrane with the primary antibody overnight at 4 °C. The antibodies used were as follows: MAZ (1:1000, 21,068-1-AP), AKT (1:1000, 10,176-2-AP), p-AKT (1:1000, 66,444-1-Ig), ERK (1:1000, 67,170-1-Ig), p-ERK (1:1000, 28,733-1-AP), p-STAT3(1:1000, ab76315), STAT3 (1:1000, 10,253-2-AP), c-MYC (1:1000, 10,828-1-AP), cyclin D1 (1:500, 26,939-1-AP), and β-actin (1:1000, sc-47778). Incubate the membrane with HRP-labeled secondary antibody (1:10,000, Rockland) at room temperature for 1 h. The membrane was treated with Immobilon™ Western chemiluminescence HRP substrate (Millipore) and detected by ECL (enhanced chemiluminescence) Fuazon Fx (Vilber Lourmat). Capture and process images with FusionCapt Advance Fx5 software (Vilber Lourmat). All experiments were repeated three times.

### Vector construction and luciferase reporter assay

Both pLKO-shMAZ and pWPI-MAZ lentiviral vectors are constructed and preserved by our laboratory. The sequence of the MAP2K2 promoter was obtained by PCR amplification with primers. And insert it into the pGL3-basic (Promega, USA) vector digested with Mlu1- and Xho1 restriction enzymes. The dual luciferase reporter gene assay was performed as previously described [23]. First, 293A cells were seeded into a 24-well plate. The MAP2K2 promoter reporter construct or the empty reporter vector was co-transfected with oeMAZ and pRL-TK into the cells and treated with AZD6244 for 24 h at the same time. The luciferase activity was measured using the Dual-Glo Luciferase Assay System (Promega, Madison, WI) with Flash and Glow reader (LB955, Berthold Technologies).

### Morphometry and histology

Fresh ccRCC and relatively normal kidney tissue are fixed in formalin. Conventional paraffin embeds the tissue. Tissue sections are 5-μm thick and stained with hematoxylin and eosin. The cross-sectional image was acquired with a Leica microscope (Leica DM6000B, Switzerland) and digitized with LAS V.4.4 (Leica).

### MTT assay

MTT [3-(4,5-dimethylthiazol-2-yl)-2,5-diphenyltetrazolium bromide] colorimetric assay was used to measured cell viability. Briefly, SW839 and Caki-1 cells were first seeded into 96-well plates. Transfect with the indicated vector or treat with AZD6244 for 24 h; then add 20 μLMTT reagent (5 mg/mL; Sigma-Aldrich) to each well, then incubate the plate for 3–4 h, and use a microplate reader (Thermo Fisher, USA) for testing.

### Colony formation assay

For the colony formation assay, 100 cultured cells/well were seeded into six-well plates and cultured for 1 week and were then fixed in a glacial acetic acid/methanol solution. Then, 0.5% crystal violet was used to stain the colonies. Colony numbers were counted under a microscope.

### ChIP assay

The chromatin immunoprecipitation (ChIP) assay was performed as previously described [24]. Briefly, Caki-1 cells were treated with formaldehyde. The cross-linked chromatin was then prepared and sonicated to an average size of 400–600 bp. The samples were diluted ten-fold and then precleared with protein A-agarose/salmon sperm DNA for 30 min at 4 °C. The DNA fragments were immunoprecipitated overnight at 4 °C with anti-MAZ or anti-IgG antibodies. After reversal of cross-linking, MAZ occupancy on the MAP2K2 promoter was examined. Results were determined by qRT-PCR. The ChIP primer sequences are summarized as follows: MAP2K2-prom-F1: GTGGTAAGGCAAGCGAGGGCG; MAP2K2-prom-R1: AGGGGAGGGGCGGCCACAAG; MAP2K2-prom-F2: GGTTCTCTCAGCCCCAGCCTG; MAP2K2-prom-R2: GGCGCCCTCGCTTGCCTTAC; MAP2K2-prom-F3: CCATCCTGGCTAACACGGTG; and MAP2K2-prom-R3: GGAGTGCAGTGGTGCGATCTC.

### Correlation analysis

The correlation analysis data between MAZ and MAP2K2 comes from RT-qPCR results and TCGA, respectively. Data from RT-qPCR result was analyzed by GraphPad Prism 7.0 software. Data from TCGA was analyzed from website of http://ualcan.path.uab.edu/analysis.html.

### Statistical analysis

Data were presented as the mean  ±  SEM. Student’s *t* test was used to analyze differences between two groups. Spearman’s correlation analysis was used to evaluate the correlation analysis. Values of P  <  0.05 were considered statistically significant. GraphPad Prism 7.0 software was used for the statistical analysis (GraphPad Software).

## Results

### The expression of MAZ increases in ccRCC tissues and leads to poor prognosis

**Fig. 1:**
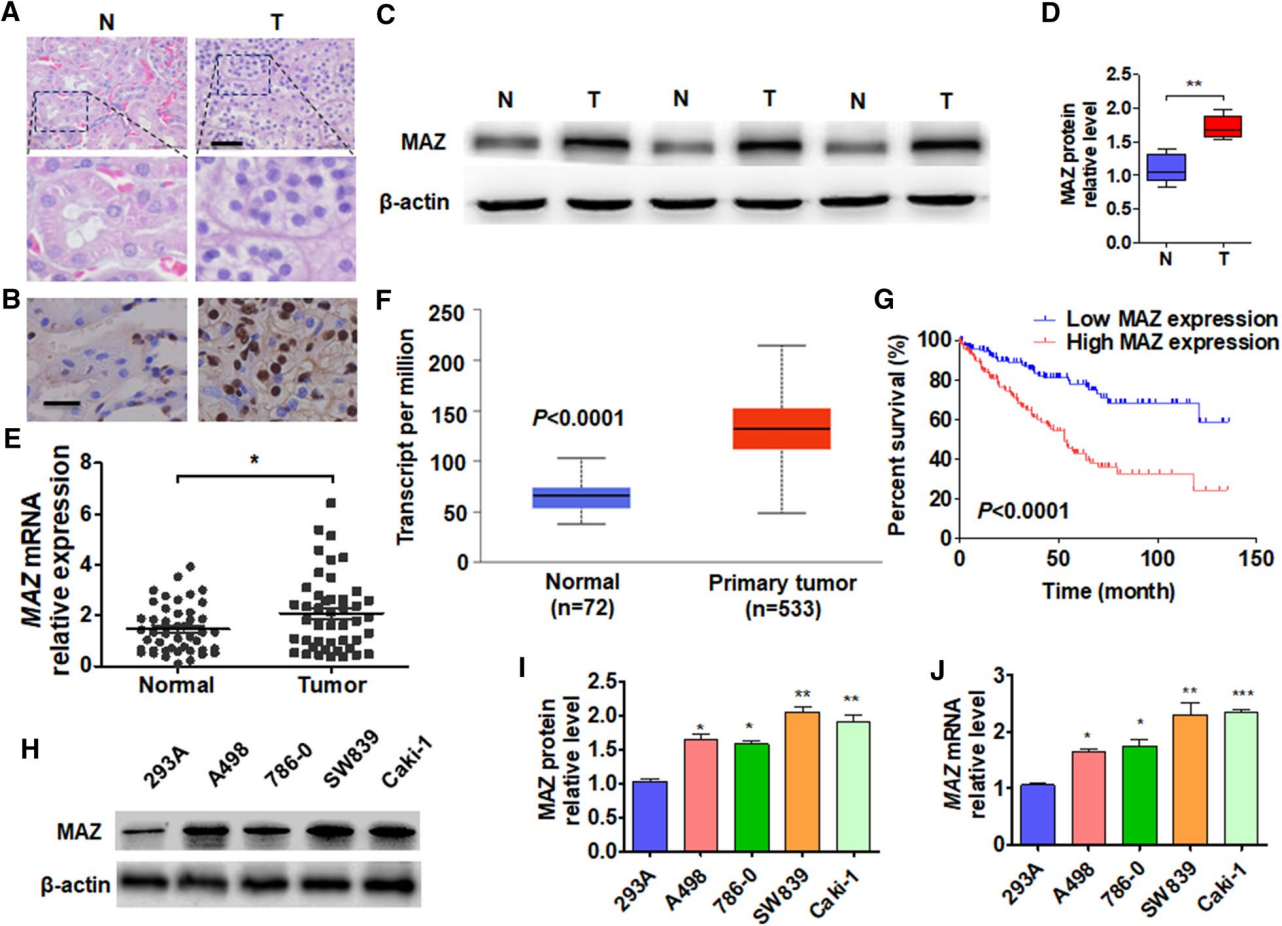
MAZ expression is increased in ccRCC and correlates with poor prognosis. **A** Hematoxylin and eosin staining of tumor (T) and normal (N) kidney tissues. Scale bar  =  50 μm. **B** Immunohistochemical staining was used to detect the expression of MAZ in tumor (T) and normal (N) kidney tissues. Scale bar  =  25 μm. **C** The levels of MAZ protein in tumor (T) and normal (N) kidney tissues were detected by Western blot. **D** Quantitative analysis of **B**. **E** The expression of MAZ mRNA was detected by qRT-PCR in tumor (T, n  =  45) and normal kidney (N, n  =  45) tissues. **(F)** MAZ mRNA levels in tumor and normal kidney tissues from the TCGA database (http://ualcan.path.uab.edu/analysis.html). **G** Kaplan–Meier analysis was used to analyze the overall survival of ccRCC patients in the TCGA database with low (n  =  130) and high (n  =  130) MAZ levels (cutoff value is 25%, http://www.oncolnc.org/). **H** Western blot analysis was used to detect the level of MAZ protein in 293A and RCC cell lines (A498, 786–0, SW839, and Caki-1). **I** Quantitative analysis of **G**. **J** qRT-PCR was performed to detect the MAZ mRNA level in the above cell lines. All data are expressed as the mean  ±  SEM from three independent experiments. *P  <  0.05, **P  <  0.01, ***P  <  0.001 vs. the corresponding controls

Studies have reported that the expression of MAZ was upregulated in prostate cancer and that it promotes tumor progression [11, 25]. However, the expression and function of MAZ in ccRCC still unclear. In order to study MAZ expression in ccRCC, we first collected the ccRCC tissues and normal kidney tissues and confirmed by HE staining (Fig. 1A). Next, we detected the MAZ expression in ccRCC and normal kidney tissues by immunohistochemical staining and western blot. The results showed that the expression of MAZ significantly increased in ccRCC tissues than that in normal kidney tissues (Fig. 1B–D). Then, we measured the mRNA expression of MAZ by using qRT-PCR in ccRCC and normal kidney tissues. Similar to protein expression, mRNA level of MAZ was significantly increased in ccRCC tissues than in normal kidney tissues (Fig. 1E). Further, we analyzed the ccRCC cases in the TCGA database and found that the expression level of MAZ mRNA in ccRCC tissues was significantly higher than that in normal kidney tissues (Fig. 1F). Using Kaplan–Meier correlation analysis we found that higher MAZ mRNA levels in ccRCC patients predict poorer overall survival in TCGA database (Fig. 1G). Furthermore, we found that the level of MAZ mRNA in ccRCC tissue was positively correlated with tumor size, but not with other clinicopathological factors, such as age, gender, and tumor grade (Table 1). Next, we detected MAZ expression in cell lines. Western blot and qRT-PCR analysis showed that the SW839 and Caki-1 cell lines expressed the highest levels of MAZ compared with the other ccRCC cell lines (Fig. 1H–J). These results suggest that MAZ upregulation may promote ccRCC progression.

**Table 1:** Clinicopathological characteristics

Characteristics	Number of patients (%)	circCOL6A3 expression		
		Low (%)	High (%)	*P* value
		26	26	
Age				
≤ 50 years	25	13 (52.00)	12 (48.00)	0.781
> 50 years	27	13 (48.15)	14 (51.85)	
Gender				
Male	37	19 (51.35)	18 (48.65)	0.760
Female	15	7 (46.67)	8 (53.33)	
Tumor size				
≤ 4 cm	20	14 (70.00)	6 (30.00)	0.023
> 4 cm	32	12 (37.50)	20 (62.50)	
T stage				
T_3_ or lower	7	4 (57.14)	3 (42.86)	0.670
T_4a_	35	16 (45.71)	19 (54.29)	
T_4b_	10	6 (60.00)	4 (40.00)	
Nods invasion				
Less than 16	33	20 (60.61)	13 (39.39)	0.044
16 or more	19	6 (31.58)	13 (68.42)	
Lauren’s classification				
Intestinal and mixed	25	12 (48.00)	13 (52.00)	0.781
Diffuse	27	14 (51.85)	13 (48.15)	

### MAZ promotes cell growth in ccRCC

**Fig. 2:**
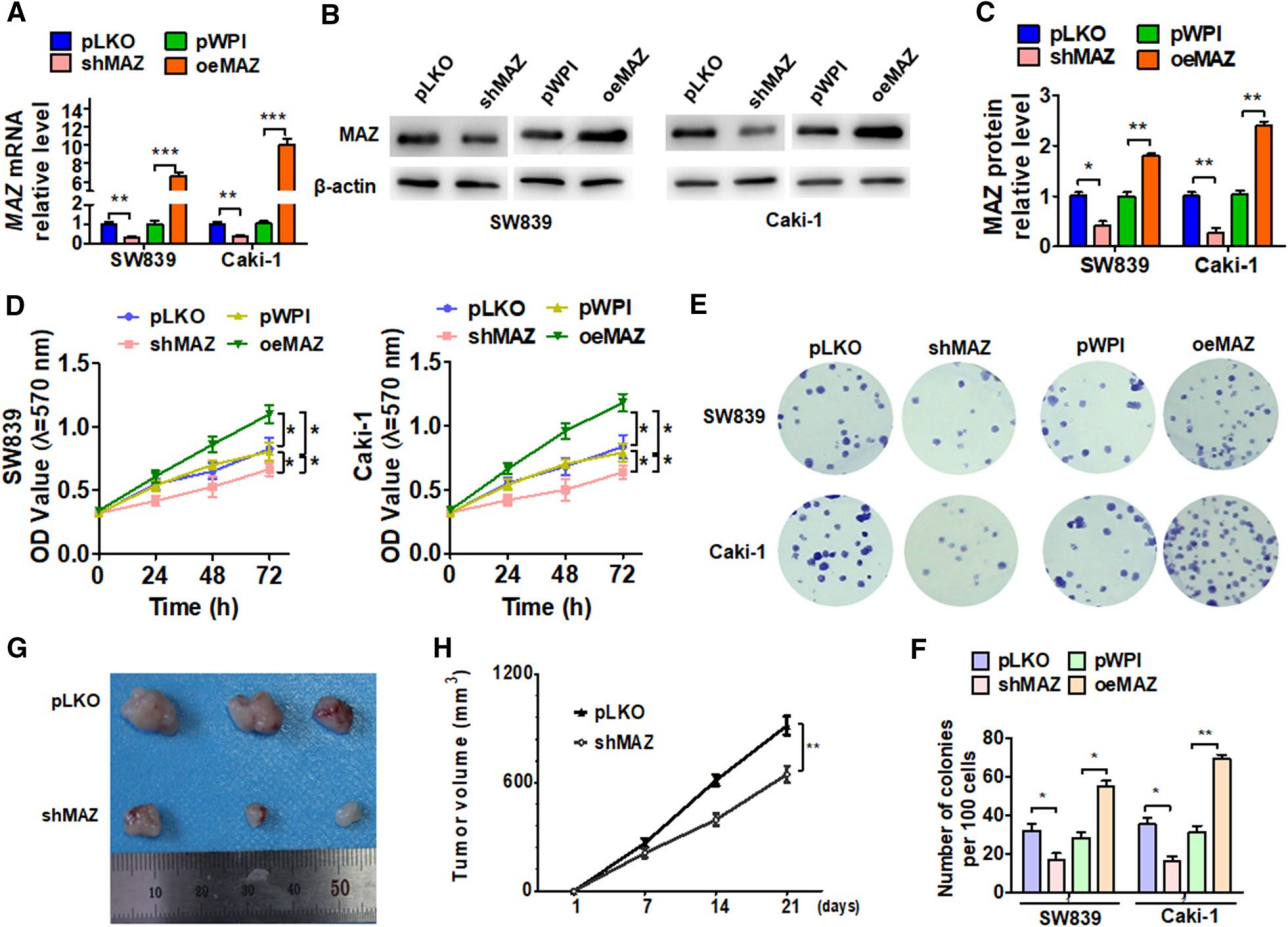
MAZ promotes ccRCC cell proliferation. **A** SW839 and Caki cells were transfected with the pKLO, shMAZ, pWPI, and oeMAZ vectors, and then qRT-PCR was used to detect the level of MAZ mRNA. **B** SW839 and Caki cells were transfected as in **A**, and the MAZ protein level was detected by Western blot. **C** Quantitative analysis of **B**. **D**, **E** Cells were prepared as in **A**, and cell viability was measured by MTT (**D**) and colony formation assays (**D–F**). **G** SW839 cells engineered to stably overexpress shMAZ or negative control (pLKO) were injected subcutaneously in 200 µl PBS/Matrigel (50:50) into the mouse forelimb. At the final time point (21 days after injection), the tumor volumes in each group were measured after resection of tumors. **H** Tumor volume was determined by direct measurement with calipers and calculated by the formula: volume  =  [(length  ×  width^2^)/2]. All data are expressed as the mean  ±  SEM from three independent experiments. *P  <  0.05, **P  <  0.01, ***P  <  0.001 vs. the corresponding controls

Studies have reported that MAZ may act as an oncogene to promote cancer progression [13, 15]. To explore the roles of MAZ in ccRCC, loss-of-function and gain-of-function experiments in vitro were performed. As shown in Fig. 2A–C, MAZ overexpression with a lentiviral vector significantly elevated the levels of MAZ mRNA and protein, whereas depletion of MAZ using shMAZ reduced MAZ expression level. Next, we detected cell viability by MTT assay, which showed that MAZ overexpression promoted growth of both SW839 and Caki-1 cells, whereas MAZ suppression inhibited proliferation of both cell lines (Fig. 2D). Similarly, a colony formation assay further confirmed these results (Fig. 2E, F). In order to explore MAZ function in vivo, we performed a mouse xenografts experiment. As showed in Fig. 2G, H, the tumor volumes were apparently smaller in nude mice implanted with MAZ-depleted cells than their corresponding control. Taken together, these data indicate that MAZ promotes the proliferation of ccRCC cells.

### The MAP2K2/ERK pathway mediates MAZ-regulated cell proliferation in vitro

**Fig. 3:**
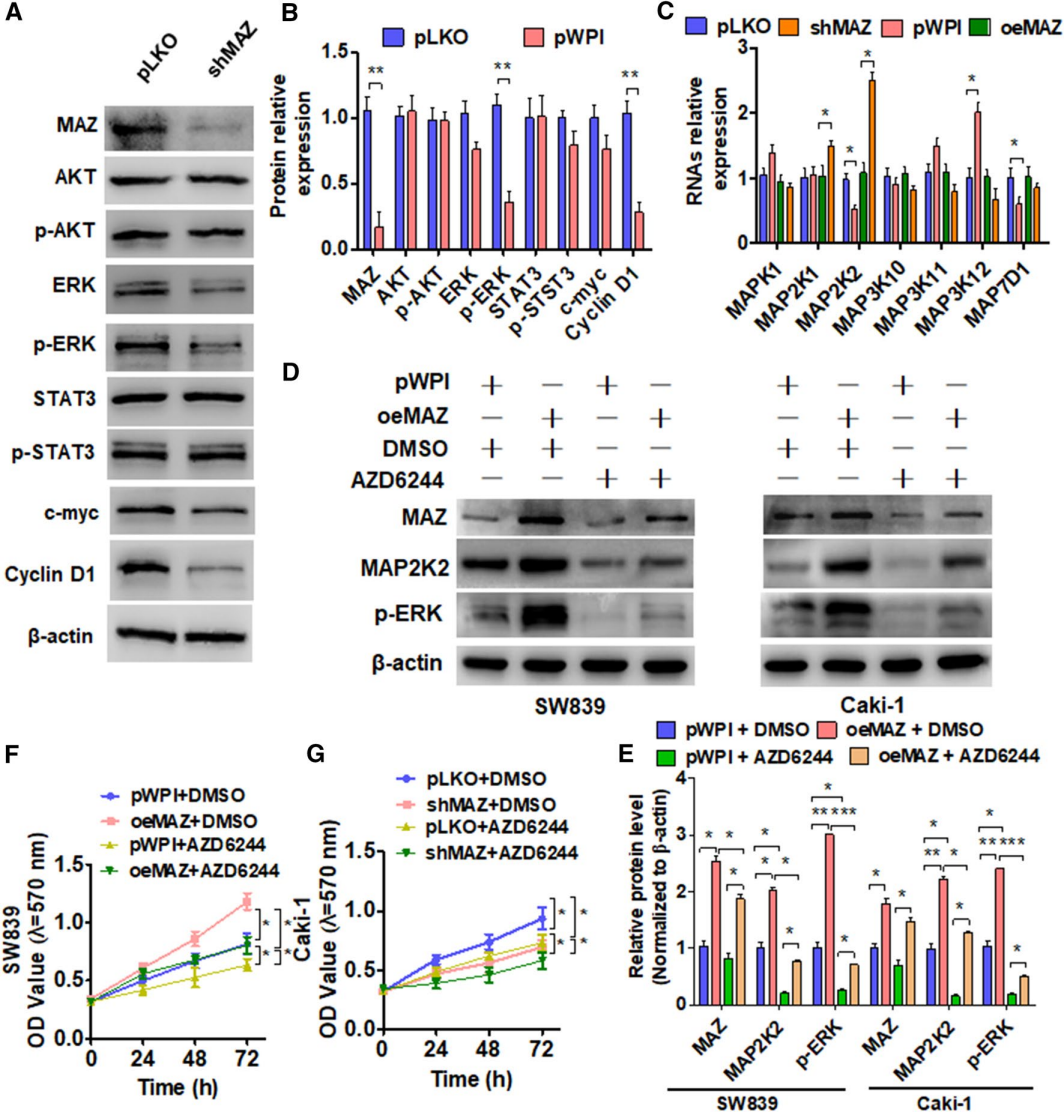
The MAP2K2/ERK pathway mediates MAZ-regulated cell proliferation. **A** Caki cells were transfected with pKLO and shMAZ, and then a Western blot analysis was used to detect the indicated protein expression. **B** Quantitative analysis of **A**. **C** Caki cells were transfected with the pKLO, shMAZ, pWPI, and oeMAZ vectors and then qRT-PCR was used to detect the mRNA levels of upstream ERK-related genes. **D** SW839 and Caki cells were transfected with the pWPI and oeMAZ vectors and were then treated with the ERK pathway inhibitor FR180204 for 24 h. Western blot was used to detect MAP2K2 and p-ERK expression. **E** Quantitative analysis of **D**. **F**, **G** Cells were transfected with the pWPI or oeMAZ (**F**) and pLKO or shMAZ (**G**) and treated with FR180204; cell viability was measured by MTT assay. All data are expressed as the mean  ±  SEM from three independent experiments. *P  <  0.05, **P  <  0.01, ***P  <  0.001 vs. the corresponding controls

To investigate which signaling pathway underlies the regulation of ccRCC cell proliferation by MAZ, we first used shRNA to knock down MAZ gene expression and then detected the expression of multiple signaling pathway molecules. The results showed that, compared with the control vector, the expression levels of AKT, p-AKT, STAT3, p-STAT3, and c-MYC were not different following MAZ knockdown. Unlike 293A cells, the protein expression of ERK, p-ERK and the downstream target gene cyclin D1 was significantly downregulated in ccRCC cells (Fig. 3A, B; Additional file 1: Figure S1). Since genes in the MAPK family are upstream of ERK, we examined which MAPK protein participates in the MAZ-regulated ERK pathway. We interfered with MAZ expression and detected its mRNA level by qRT-PCR. As shown in Fig. 3C, only MAP2K2 was downregulated in MAZ knockdown cells, whereas MAP2K2 was elevated in MAZ-overexpressing Caki-1 cells. However, MAZ does not affect the KRAS signal pathway (Additional file 1: Figure S2). To confirm that MAP2K2 mediates the MAZ-regulated ERK pathway, we overexpressed MAZ and then treated cells with a MAPK inhibitor (AZD6244). The results showed that MAZ overexpression increased the levels of MAP2K2 and p-ERK proteins, whereas AZD6244 treatment reversed MAP2K2 and p-ERK protein expression promoted by MAZ (Fig. 3D, E). Furthermore, MTT analysis showed that MAZ overexpression promoted proliferation of SW839 cells, whereas AZD6244-induced suppression of MAZ enhanced cell proliferation (Fig. 3F). Similarly, AZD6244 treatment enhanced the MAZ-depleted inhibition of Caki-1 cell growth (Fig. 3G). These findings support the finding that the MAP2K2/ERK pathway underlies the regulation of ccRCC cell proliferation by MAZ.

### MAP2K2 is upregulated in ccRCC tissues and the expression is positively correlated with MAZ

**Fig. 4:**
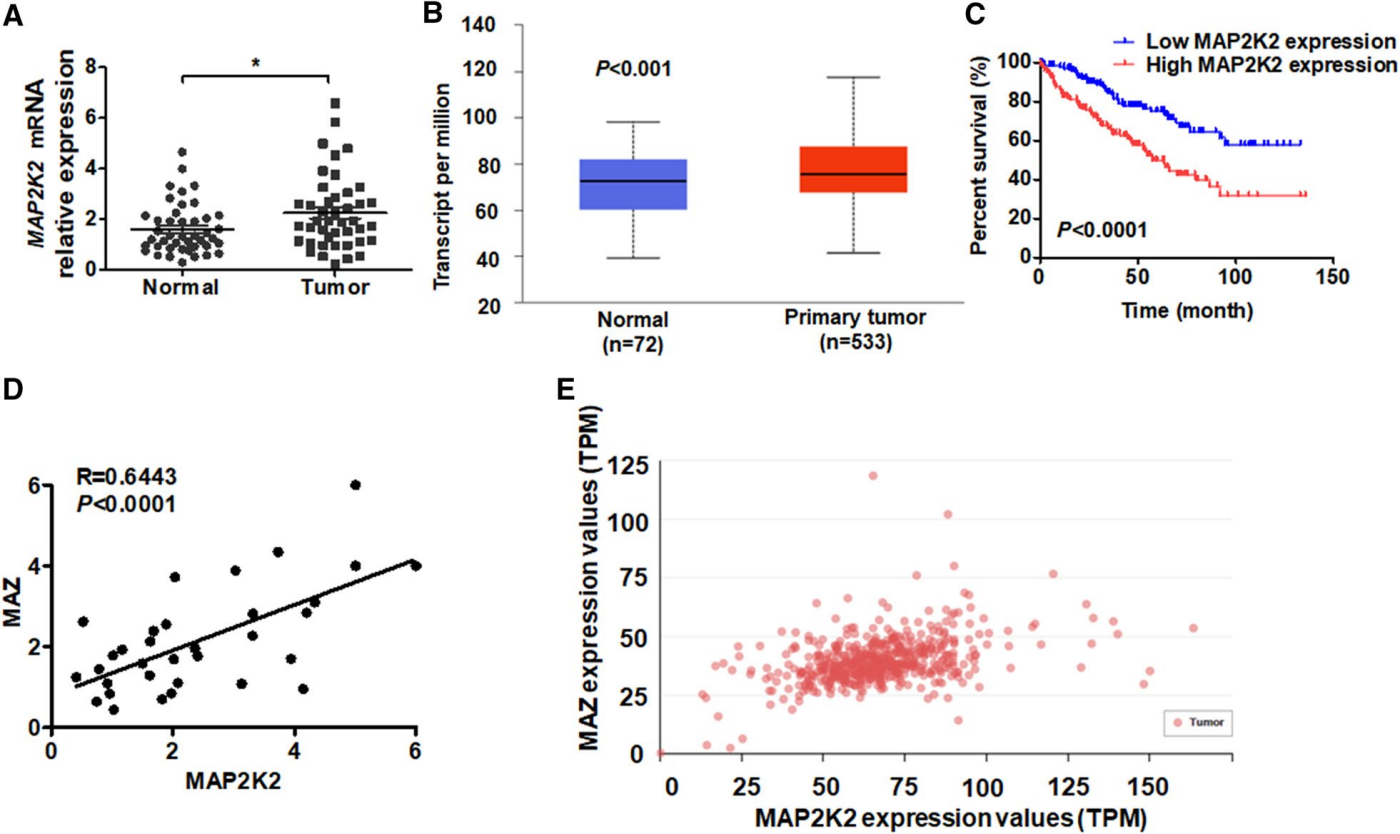
MAP2K2 is upregulated in ccRCC and is positively correlated with MAZ. **A** The MAP2K2 mRNA level was detected by qRT-PCR in tumor (T, n  =  45) and normal kidney (N, n  =  45) tissues. **B** The expression of MAP2K2 mRNA was analyzed in the TCGA database (http://ualcan.path.uab.edu/analysis.html). **C** Kaplan–Meier analysis was used to analyze the overall survival of ccRCC patients from the TCGA database with low (n  =  130) and high (n  =  130) MAP2K2 levels (http://www.oncolnc.org/). **D**, **E** The correlation between MAZ and MAP2K2 mRNA expression in PCa tissues was analyzed by Pearson correlation analysis of our clinical data (R  =  0.6443, P  <  0.0001). **D** or other data published in the TCGA database **E** (http://ualcan.path.uab.edu/analysis.html). All data are expressed as the mean  ±  SEM from three independent experiments. *P  <  0.05, **P  <  0.01 vs. the corresponding controls

In order to detect the expression of MAP2K2 in ccRCC, we first measured the level of MAP2K2 in ccRCC tissues and normal kidney tissues by qRT-PCR. The levels of MAP2K2 mRNA were significantly eleved in ccRCC tissues than that in the normal kidney tissues (Fig. 4A). Next, we examined MAP2K2 mRNA expression in ccRCC cases from the TCGA database. The results confirmed that MAP2K2 mRNA expression markedly higher in ccRCC tissues than in normal kidney tissues (Fig. 4B). In addition, the database from TCGA also confirmed that the higher levels of MAZ mRNA in patients with ccRCC were associated with poor overall survival (Fig. 4C). Next, we analyzed the correlation between MAZ and MAP2K2 mRNA in ccRCC tissues and TCGA data. We found that MAP2K2 was positively correlated with MAZ in ccRCC tissues (Fig. 4D, E). Collectively, these results indicate that MAP2K2 is positively correlated with MAZ, which is also upregulated in ccRCC.

### MAZ binds to the MAP2K2 promoter and regulates its expression

**Fig. 5:**
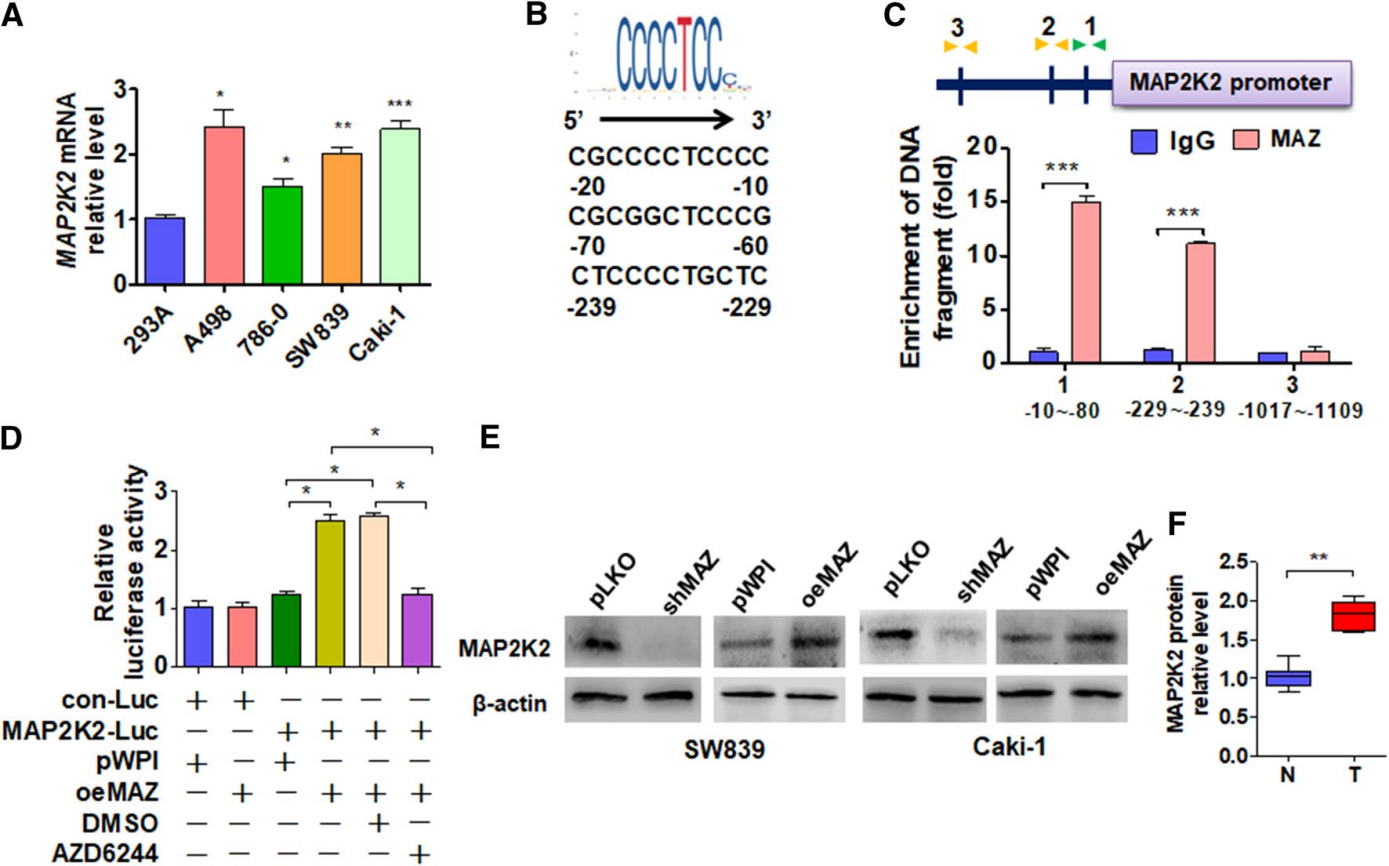
MAZ promotes the transcription of MAP2K2 in ccRCC cells. **A** qRT-PCR was used to detect MAP2K2 expression in the above indicated cell lines. **B** Potential binding site of MAZ in the MAP2K2 promotor. **C** ChIP-qPCR was used to measure the binding of the transcription factor MAZ to the MAP2K2 promoter region in Caki-1 cells. **D** Cells were co-transfected with the MAP2K2 promoter-luciferase reporter and pWPI or oeMAZ, transfected with the oeMAZ vector and treated simultaneously with FR180204, and then luciferase reporter assays were performed. **E** SW839 and Caki cells were transfected with the pKLO, shMAZ, pWPI, and oeMAZ vectors, and then Western blot was performed to detect the MAP2K2 protein level. **F** Quantitative analysis of **E**. All data are expressed as the mean  ±  SEM from three independent experiments. *P  <  0.05, **P  <  0.01, ***P  <  0.001 vs. the corresponding controls

Studies have shown that MAZ acts as a transcription factor that regulates gene expression [26]. First, we detected MAZ expression in cell lines and found that MAP2K2 was also highly expressed in SW839 and Caki-1 cells (Fig. 5A). To investigate whether MAZ regulates MAP2K2 expression by transcription regulation, we predicted the potential binding site of MAZ within the 2-kb 5′-promoter region of MAP2K2 using the Ensembl and PROMO 3.0 websites. We found that there are three potential MAZ binding sites (Fig. 5B). ChIP analysis showed that MAZ mainly binds in the region of -10 to  − 80 bp or  − 229 to  − 239 upstream of the transcription start site of the MAP2K2 promoter (Fig. 5C). Similarity, the luciferase assay demonstrated that overexpression of MAZ significantly elevated the luciferase activity while AZD6244 treatment depressed it (Fig. 5D). Western blot also verified that MAZ overexpression increased MAP2K2 but not MEK1 (MAP2K1) protein levels (Fig. 5E, F, Additional file 1: Figure S3). These findings indicate that MAZ directly promotes MAP2K2 transcription.

### The MAZ/MAP2K2/ERK pathway plays a critical role in ccRCC cell growth

**Fig. 6:**
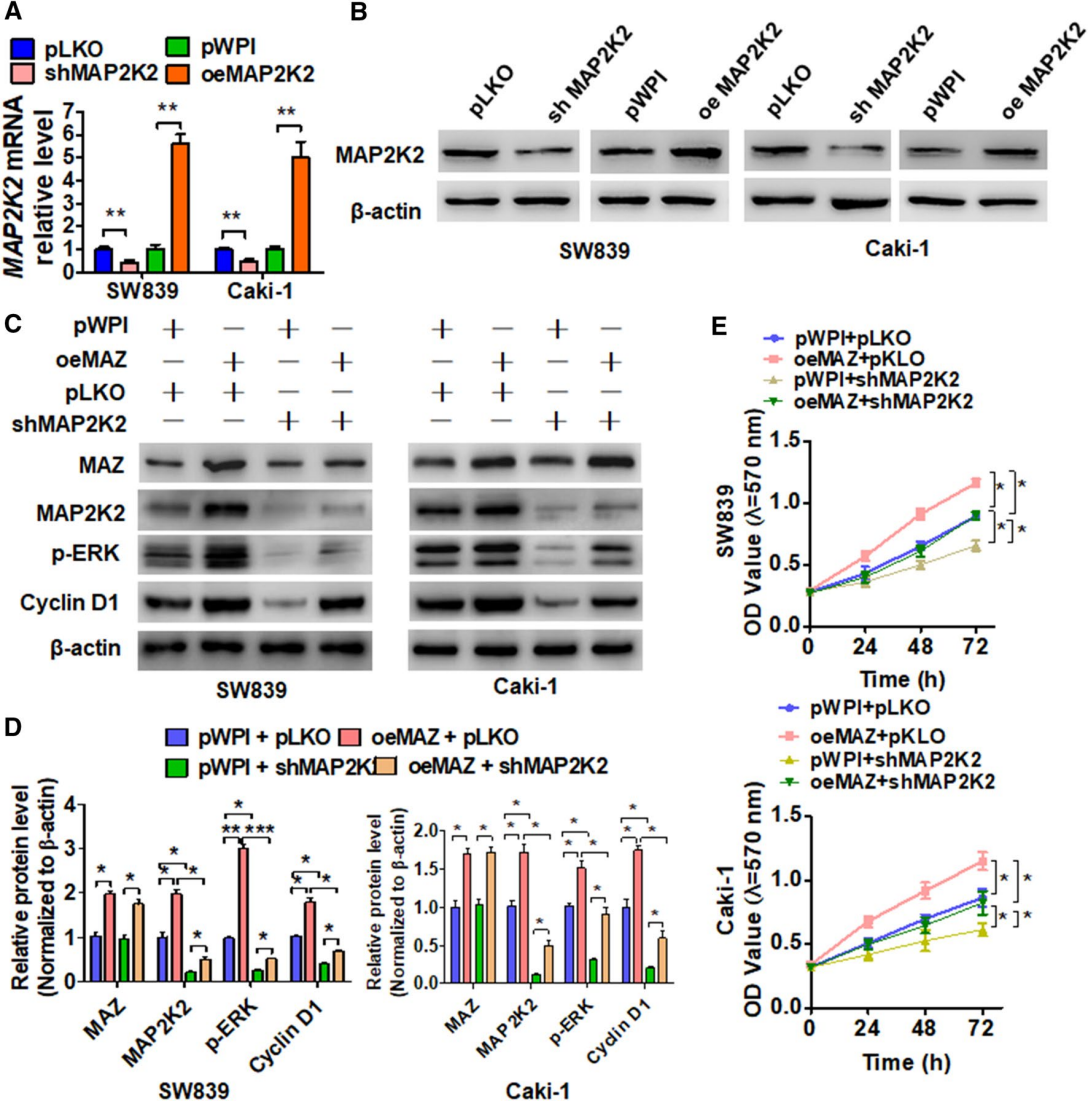
The MAZ/MAP2K2/ERK axis promotes ccRCC progression. **A** SW839 and Caki cells were transfected with the pKLO, shMAP2K2, pWPI, and oeMAP2K2 vectors and then subjected to qRT-PCR to determine the MAP2K2 mRNA level. **B** SW839 and Caki cells were transfected as in **A**, and the MAP2K2 protein level was detected by Western blot. **C** SW839 and Caki cells were transfected with the indicated vectors and then Western blot was performed to determine the protein levels of MAP2K2, p-ERK, and cyclin D1. **D** Quantitative analysis of **C**. **E** Cells were prepared as in **C**, and cell viability was measured by MTT assay. All data are expressed as the mean  ±  SEM from three independent experiments. *P  <  0.05, **P  <  0.01, ***P  <  0.001 vs. the corresponding controls

To explored the functions of MAP2K2 in ccRCC, we performed several in vitro loss-of-function and gain-of-function experiments. The results showed that MAP2K2 overexpression significantly elevated the protein levels and mRNA of MAP2K2, whereas depletion of MAP2K2 using shMAP2K2 reduced MAP2K2 expression (Fig. 6A, B). To elucidate whether the MAZ/MAP2K2/ERK pathway is involved in ccRCC cell growth, we conducted a rescue experiment. First, SW839 and Caki-1 cells that overexpressed MAZ or that lacked MAP2K2 expression or both were established. Western blot was used to determine the expression of these proteins. As shown in Fig. 6C, D, MAZ overexpression promoted MAP2K2, p-ERK, and cyclin D1 expression, whereas simultaneous MAP2K2 knockdown significantly depressed MAZ-promoted protein expression. Furthermore, MTT analysis confirmed that MAZ overexpression promoted growth of both SW839 and Caki-1 cells, whereas MAP2K2 suppression reversed MAZ-promoted proliferation in both cell lines (Fig. 6E). Together, these findings indicate that the MAZ/MAP2K2/ERK pathway promotes ccRCC cell growth (Additional file 2: Figure S1–8).

## Discussion

In this study, we explored the biological role of the MAZ/MAP2K2/ERK pathway in the modulation of ccRCC oncogenesis. First, it was found from the TCGA database and clinical samples that the expression of MAZ in ccRCC tissues was significantly increased, and the increase in MAZ in patients was associated with a poor prognosis. Secondly, MAZ proved to be an oncogene because it promotes the proliferation of ccRCC. Third, MAZ positively regulates the expression of MAP2K2. Mechanistically, MAZ was bound to the MAP2K2 promoter and promoted its transcription. MAP2K2 upregulation enhanced ERK phosphorylation and activated the MEK/ERK signaling cascade. Our findings suggest that MAZ/MAP2K2/ERK signaling plays a role in growth promotion in ccRCC.

There are four different MAPK signal cascades in mammals, including extracellular signal-regulated kinase or MAPK, c-Jun N-terminal kinase, p38 and ERK5 [27]. Among them, the ERK/MAPK pathway is considered a “classic” MAPK signal level[28]. The MEK/ERK (MAPK) signal cascade plays an important role in regulating the basic functions of cells, such as growth, survival and differentiation [17]. MEK includes MEK1 and MEK2 (MAP2K2), they have 79% amino acid homology, and they both have the ability to phosphorylate downstream molecule ERK [16]. Although MEK2 plays an important role in the MAPK signaling cascade, its expression in ccRCC remains to be explored. In this study, we found that MAP2K2 (MEK2) expression was upregulated in ccRCC tissues. MAP2K2 upregulation promoted ccRCC cell growth by activating ERK phosphorylation. Further experiments showed that MAZ-promoted MAP2K2 expression, but not that of other MAPK kinases, through which it regulated cell proliferation. In order to investigate whether MAZ participated in ERK pathway in 293A cell lines, we knocked down the MAZ and detected those pathways signal molecular. Our result showed that depletion of MAZ reduced the p-STAT3 not p-ERK level in 293A cell lines. These results indicated that the activation of the MAZ-dependent ERK pathway was essential for renal cancer cell lines.

Both MEK1 and MEK2 activate Erk/MAP kinase related to cell growth and differentiation [29]. Studies on mice have shown that Mek1^(−/−)^ embryos die due to placental defects, while Mek2^(−/−)^ mice survive with normal life span and fertility. This indicates that MEK1 has functions that are not shared by MEK2. However, most Mek1 ^(+/−)^ Mek2 ^(+/−)^ embryos also died of placental defects, indicating that both Mek genes contribute to placental development [30]. In addition, MEK2 may be the main Erk/MAP kinase activator during development, while MEK1 may play a role in proliferation or mitogenic response [31]. Studies have shown that the regulation of the expression of multiple genes during the development of tumors is similar to that of the embryonic development of the body [32, 33]. Our research also shows that MEK2 also plays an important role in the proliferation and migration of ccRCC cells. Studies have reported that treatment of cells with AZD6244 for a short time reduced the phosphorylation level of MEK1/2 instead of regulating the protein expression of MEK1/2 [34, 35]. However, our results showed that AZD6244 decreased MEK2 protein level (Fig. 3D). The reason may be that AZD6244 interferes with the transcription activity of a variety of transcription factors, such as c-myc, E2F1, etc., by inhibiting the MEK/ERK signaling pathway. These transcription factors may downregulate the expression of MEK2 through transcriptional regulation.

MAZ is upregulated in many types of cancers and is widely involved in the occurrence, development and metastasis of tumors [11–13]. Recent studies have found that the upregulation of MAZ promotes the proliferation and metastasis of prostate cancer by promoting androgen receptor expression [25]. In addition, MAZ plays a key role in promotion of prostate cancer bone metastasis via transcriptional activation of the KRas/RalGEFs pathway [11]. MAZ, which is a downstream molecular of the Cyr61/CCN1, promotes pancreatic cancer cell invasion via CRAF-ERK signaling [12]. However, the clinical significance and biological role of MAZ in ccRCC still unclear. In the present study, the results showed that MAZ was elevated in ccRCC tissues than in normal kidney tissues and that MAZ expression was positively correlated with poor overall survival of ccRCC patients. Interestingly, MAZ upregulation activated the ERK signaling cascade. This discovery stumulated our interest in further exploring the mechanism that the dramatic differential expression of MAZ between normal and cancerous tissues, in which signaling factors were shown to be regulated by MAZ. Forthermore, the finding revealed that MAZ upregulation promoted MAP2K2 expression, whereas MAZ silencing inhibited MAP2K2 gene expression. Our results further demonstrate that MAZ-promoted ccRCC cell proliferation via transcriptional activation of MAP2K2/ERK signaling. Thus, these findings suggest that MAZ functions as an oncogene in ccRCC.

In summary, our results reveal that upregulation of MAZ activates the MAP2K2/ERK signaling pathway, which further promotes cell growth in ccRCC. These data provide evidence that the MAZ/MAP2K2/ERK signaling axis plays a role in ccRCC progression. Therefore, a comprehensive understanding of the mechanism that activates the MAPK signaling pathway will help develop effective therapeutic strategies to inhibit the progression of ccRCC.

## Supplementary Information

**Additional file 1: Figure S1. **MAZ participate in STAT3 signal pathway in 293A cell. 293A cell transfected with shMAZ or pLKO vector, and then western blot detected several pathways signal molecular. **Figure S2. **SW839 cell transfected with oeMAZ or pWPI vector and then Raf1 or p-Raf1 protein level was detected by Western blot. **Figure S3. **SW839 cell transfected with indicated vectors and Western blot detected MEK1 protein expression.

**Additional file 2: Figure S1.** The original immunoblots of Fig. 1C. **Figure S2.** The original immunoblots of Fig. 1G. **Figure S3.** The original immunoblots of Fig. 2B. **Figure S4.** The original immunoblots of Fig. 3A. **Figure S5.** The original immunoblots of Fig. 4D. **Figure S6.** The original immunoblots of Fig. 5E. **Figure S7.** The original immunoblots of Fig. 6B. **Figure S8.** The original immunoblots of Fig. 6C.

## Data Availability

Not applicable.
