# Supplementary material for: Myc-associated zinc-finger protein promotes clear cell renal cell carcinoma progression through transcriptional activation of the MAP2K2-dependent ERK pathway
Source: Cancer Cell Int. 2021 Jun 28;21:323. doi: 10.1186/s12935-021-02020-9 (PMC8240279; doi:10.1186/s12935-021-02020-9)
Supplement: Supplementary file 1 — Additional file 1: Figure S1. MAZ participate in STAT3 signal pathway in 293A cell. 293A cell transfected with shMAZ or pLKO vector, and then western blot detected several pathways signal molecular. Figure S2. SW839 cell transfected with oeMAZ or pWPI vector and then Raf1 or p-Raf1 protein level was detected by Western blot. Figure S3. SW839 cell transfected with indicated vectors and Western blot detected MEK1 protein expression. [file 12935_2021_2020_MOESM1_ESM.docx]

**Additional file 1**

**
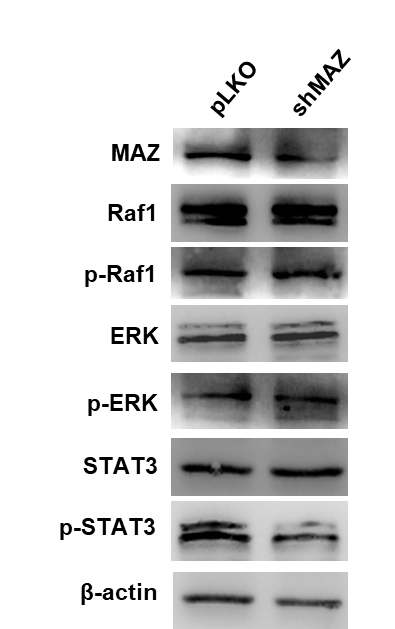
**

**Figure S1** MAZ participate in STAT3 signal pathway in 293A cell. 293A cell transfected with shMAZ or pLKO vector, and then western blot detected several pathways signal molecular.

**
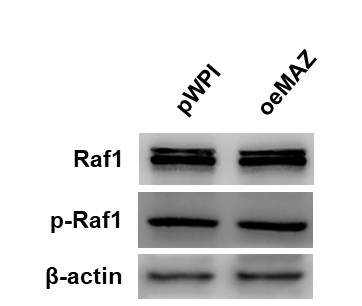
**

**Figure S2** SW839 cell transfected with oeMAZ or pWPI vector and then Raf1 or p-Raf1 protein level was detected by Western blot.

**
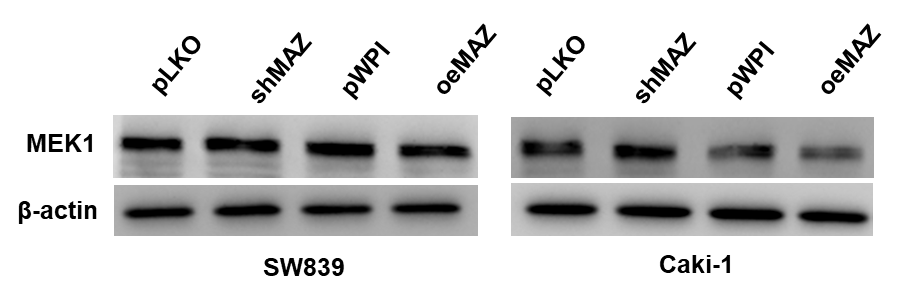
**

**Figure S3** SW839 cell transfected with indicated vectors and Western blot detected MEK1 protein expression.
