# Supplementary material for: Myc-associated zinc-finger protein promotes clear cell renal cell carcinoma progression through transcriptional activation of the MAP2K2-dependent ERK pathway
Source: Cancer Cell Int. 2021 Jun 28;21:323. doi: 10.1186/s12935-021-02020-9 (PMC8240279; doi:10.1186/s12935-021-02020-9)
Supplement: Supplementary file 2 — Additional file 2: Figure S1. The original immunoblots of Fig. 1C. Figure S2. The original immunoblots of Fig. 1G. Figure S3. The original immunoblots of Fig. 2B. Figure S4. The original immunoblots of Fig. 3A. Figure S5. The original immunoblots of Fig. 4D. Figure S6. The original immunoblots of Fig. 5E. Figure S7. The original immunoblots of Fig. 6B. Figure S8. The original immunoblots of Fig. 6C. [file 12935_2021_2020_MOESM2_ESM.docx]

**kDa**

**34**

**43**

**β-actin**


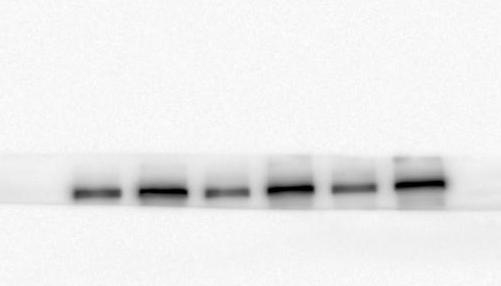


**kDa**

**43**

**55**

**MAZ**

**Supplementary Figure 1.** The original immunoblots of Figure 1C.


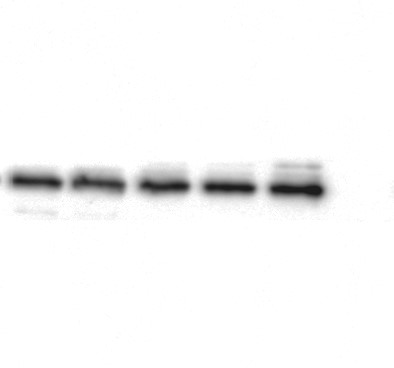





**kDa**

**43**

**34**

**β-actin**


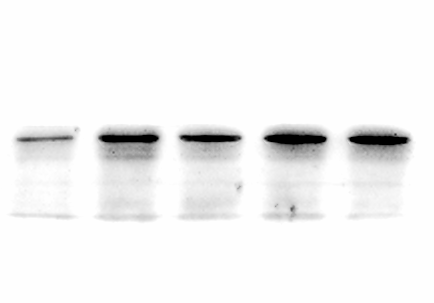


**kDa**

**43**

**55**

**MAZ**

**Supplementary Figure 2.** The original immunoblots of Figure 1G.


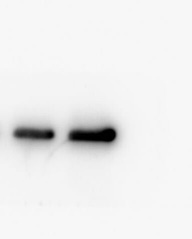


**kDa**


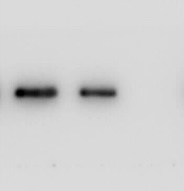


**kDa**

**55**

**55**

**43**

**43**

**MAZ**

**MAZ**


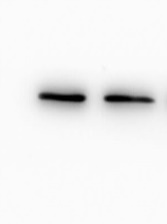

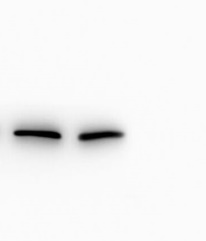


**kDa**

**34**

**43**

**kDa**

**34**

**43**

**β-actin**

**β-actin**

**kDa**


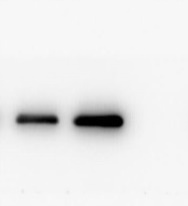


**kDa**


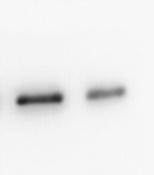


**55**

**55**

**43**

**43**

**MAZ**

**MAZ**


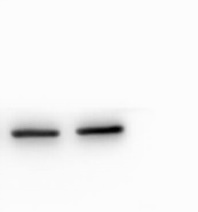


**kDa**

**34**

**43**

**kDa**


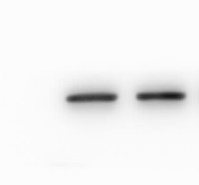


**34**

**43**

**β-actin**

**β-actin**

**Supplementary Figure 3.** The original immunoblots of Figure 2B.

**kDa**

**43**

**55**

**kDa**

**55**

**75**

**kDa**

**55**

**75**


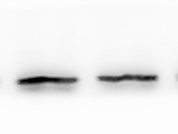

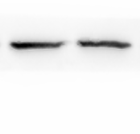

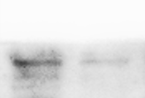


**AKT**

**MAZ**

**p-AKT**

**kDa**


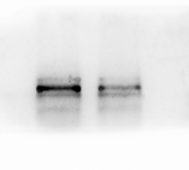


**kDa**

**kDa**


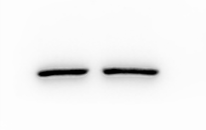

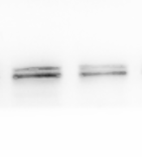


**100**

**43**

**43**

**75**

**34**

**34**

**STAT3**

**p-ERK**

**ERK**

**75**

**100**

**kDa**

**kDa**

**kDa**

**kDa**


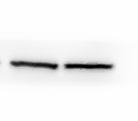

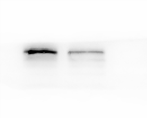

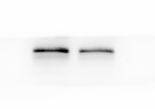

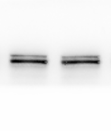


**43**

**34**

**75**

**34**

**43**

**55**

**β-actin**

**Cyclin D1**

**c-myc**

**p-STAT3**

**Supplementary Figure 4.** The original immunoblots of Figure 3A.


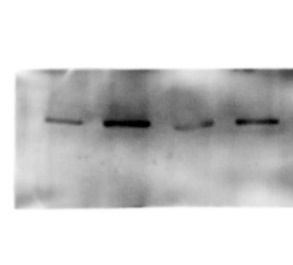


**kDa**

**55**

**43**

**MAZ**

**kDa**


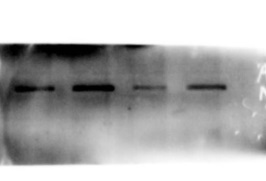


**55**

**43**

**MAZ**

**kDa**

**55**

**43**

**MAP2K2**

**kDa**

**55**

**43**

**MAP2K2**


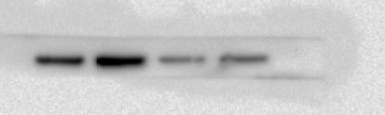


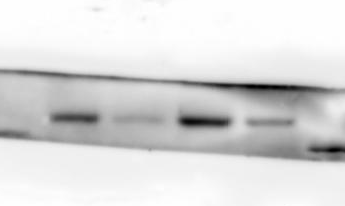


**kDa**

**43**

**34**

**p-ERK**

**kDa**

**43**

**34**

**p-ERK**


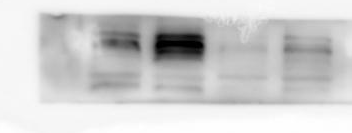


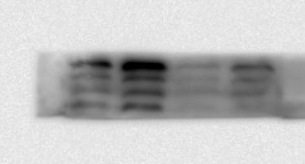


**kDa**

**43**

**34**

**β-actin**


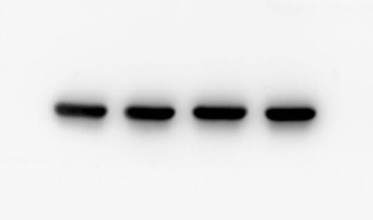


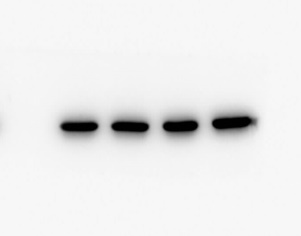


**kDa**

**43**

**34**

**β-actin**

**Supplementary Figure 5.** The original immunoblots of Figure 4D.

**kDa**


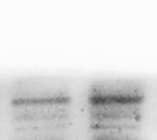


**kDa**

**55**

**55**


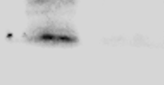


**43**

**43**

**MAP2K2**

**MAP2K2**

**kDa**

**34**

**43**

**kDa**


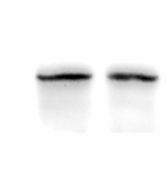

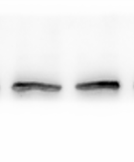


**34**

**43**

**β-actin**

**β-actin**

**kDa**


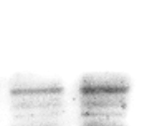


**kDa**

**55**

**55**


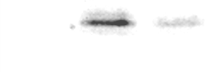


**43**

**43**

**MAP2K2**

**MAP2K2**


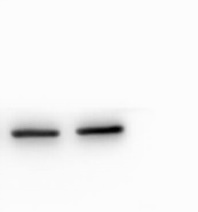


**kDa**

**34**

**43**

**kDa**


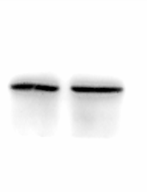


**34**

**43**

**β-actin**

**β-actin**

**Supplementary Figure 6.** The original immunoblots of Figure 5E.

**kDa**

**kDa**


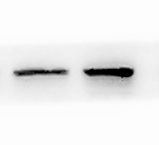

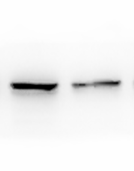


**55**

**55**

**43**

**43**

**MAP2K2**

**MAP2K2**

**kDa**

**34**

**43**

**kDa**


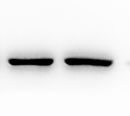

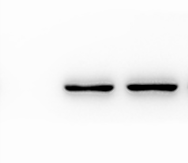


**34**

**43**

**β-actin**

**β-actin**

**kDa**


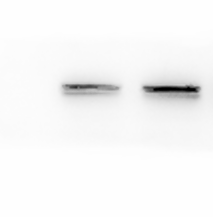


**kDa**


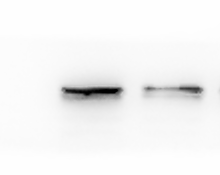


**55**

**55**

**43**

**43**

**MAP2K2**

**MAP2K2**

**kDa**

**43**

**kDa**


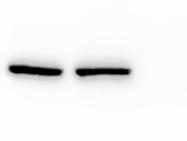

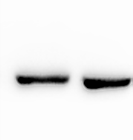


**43**

**34**

**34**

**β-actin**

**β-actin**

**Supplementary Figure 7.** The original immunoblots of Figure 6B.


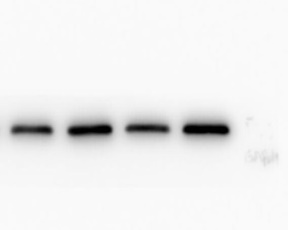

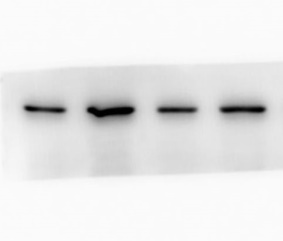


**kDa**

**55**

**43**

**MAZ**

**kDa**

**55**

**43**

**MAZ**


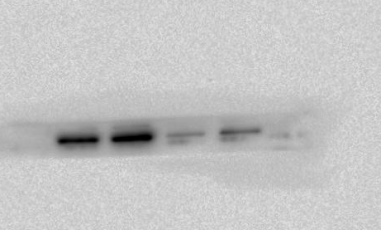


**kDa**

**55**

**43**

**MAP2K2**

**kDa**

**55**

**43**

**MAP2K2**


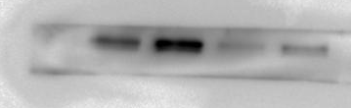


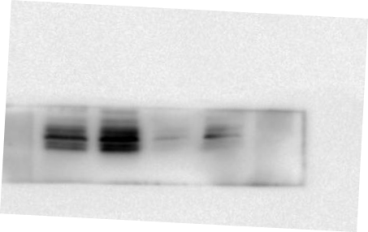


**kDa**

**43**

**34**

**p-ERK**

**kDa**

**43**

**34**

**p-ERK**


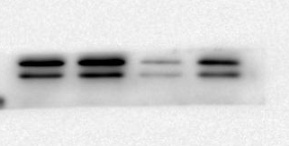


**kDa**

**43**

**34**

**Cyclin D1**


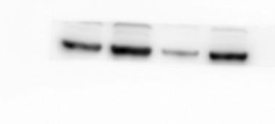


**kDa**

**43**

**34**

**Cyclin D1**


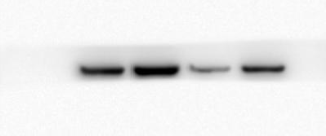


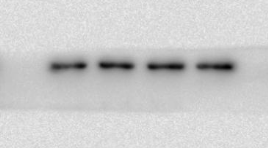


**kDa**

**43**

**34**

**β-actin**

**kDa**

**43**

**34**

**β-actin**


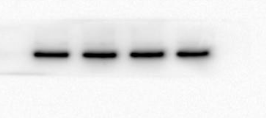


**Supplementary Figure 8** The original immunoblots of Figure 6C.
